# Supplementary material for: Exploring mechanical work changes in controlled ankle motion (CAM) boot walking: The effects of gait speed and shoe levelling
Source: PLoS One. 2025 Apr 29;20(4):e0321978. doi: 10.1371/journal.pone.0321978 (PMC12040088; doi:10.1371/journal.pone.0321978)
Supplement: S1 Table — Total mechanical work and joint mechanical work for the ankle, knee, and hip at each speed and in each footwear condition. ANOVA statistics presented in right-hand column. (DOCX) [file pone.0321978.s001.docx]

**Table S1. Data corresponded to manuscript Figures 1-4. Total mechanical work and joint mechanical work for the ankle, knee, and hip at each speed and in each footwear condition. ANOVA statistics presented in right-hand column.**

| **Parameter** | **Limb** | **Speed** | **NORM** | **BOOT** | **EVEN** | ***ANOVA*** |  |
| --- | --- | --- | --- | --- | --- | --- | --- |
| **Figure 1** | | | | | | |  |
| **W_tot_  (J∙kg^-1^)** | ***Left**** | PWS | 1.37 ± 0.28 | 1.27 ± 0.27 | 1.36 ± 0.18 | N/A |  |
|  |  | 3 km/h  4 km/h  5 km/h | 0.94 ± 0.20  1.20 ± 0.23  1.45 ± 0.20 | 0.86 ± 0.17  1.11 ± 0.19  1.35 ± 0.18 | 0.98 ± 0.17  1.26 ± 0.17  1.51 ± 0.19 | **Condition:** BOOT < NORM (*p* = 0.047) & EVEN (*p* < 0.001)  **Speed:** All speeds different to all other speeds (*p* < 0.001) |  |
|  | ***Right*** | PWS | 1.37 ± 0.25 | 0.95 ± 0.22 | 0.93 ± 0.19 | BOOT (*p* < 0.001) & EVEN (*p* = 0.001) < NORM |  |
|  |  | 3 km/h  4 km/h  5 km/h | 1.00 ± 0.29  1.25 ± 0.32  1.47 ± 0.26 | 0.70 ± 0.19  0.86 ± 0.21  1.00 ± 0.14 | 0.63 ± 0.13  0.81 ± 0.14  0.99 ± 0.16 | **Condition:** BOOT & EVEN < NORM (*p* < 0.001)  **Speed:** All speeds different to all other speeds (*p* < 0.001)  **Interaction:** Condition reacted to speeds similarly (*p* ≤ 0.024) |  |
| **Figure 2** | | | | | | |  |
| **W_ankle_  (J∙kg^-1^)** | ***Left**** | PWS | 0.43 ± 0.05 | 0.40 ± 0.06 | 0.40 ± 0.07 | **Condition:** BOOT < NORM (*p* = 0.011) |  |
|  |  | 3 km/h  4 km/h  5 km/h | 0.33 ± 0.07  0.41 ± 0.08  0.45 ± 0.08 | 0.33 ± 0.06  0.39 ± 0.07  0.42 ± 0.06 | 0.30 ± 0.06  0.37 ± 0.05  0.41 ± 0.07 | **Speed:** 3 km/h < 4 & 5 km/h (*p* < 0.001);  4 km/h < 5 km/h (*p* = 0.007) |  |
|  | ***Right*** | PWS | 0.38 ± 0.13 | 0.05 ± 0.02 | 0.06 ± 0.01 | **Condition:** BOOT & EVEN < NORM (*p* < 0.001) |  |
|  |  | 3 km/h  4 km/h  5 km/h | 0.34 ± 0.07  0.40 ± 0.11  0.44 ± 0.10 | 0.05 ± 0.01  0.05 ± 0.02  0.05 ± 0.02 | 0.04 ± 0.01  0.05 ± 0.01  0.06 ± 0.01 | **Condition:** BOOT & EVEN < NORM (*p* < 0.001)  **Speed:** 3 km/h < 4 & 5 km/h (*p* < 0.001);  4 km/h < 5 km/h (*p* = 0.008)  **Interaction:** NORM was different at all speeds (*p* ≤ 0.005) but BOOT & EVEN were not |  |
| **W_ankle_  (% W_tot_)** | ***Left**** | PWS | 32 ± 5 | 32 ± 5 | 28 ± 3 | **Condition:** EVEN < BOOT (*p* = 0.037) |  |
|  |  | 3 km/h  4 km/h  5 km/h | 36 ± 5  34 ± 3  31 ± 3 | 39 ± 6  36 ± 3  31 ± 4 | 31 ± 6  29 ± 4  27 ± 4 | **Condition:** EVEN < NORM (*p* = 0.001) & BOOT (*p* < 0.001)  **Speed:** 5 km/h < 3 km/h (*p* = 0.012) & 4 km/h (*p* = 0.003)  **Interaction:** NORM: 5 km/h < 3 km/h (*p* = 0.049) & 4 km/h (*p* = 0.039); OSS: different at all speeds (*p* ≤ 0.007); EVEN: 5 km/h < 4 km/h (*p* = 0.004) |  |
|  | ***Right*** | PWS | 28 ± 9 | 6 ± 1 | 6 ± 2 | **Condition:** BOOT & EVEN < NORM (*p* < 0.001) |  |
|  |  | 3 km/h  4 km/h  5 km/h | 34 ± 6  32 ± 3  30 ± 3 | 7 ± 2  6 ± 1  6 ± 1 | 6 ± 2  6 ± 2  6 ± 1 | **Condition:** BOOT & EVEN < NORM (*p* < 0.001)  **Speed:** 5 km/h < 3 km/h (*p* = 0.032) & 4 km/h (*p* = 0.001) |  |
| **Figure 3** | | | | | | | |
| **W_knee_  (J∙kg^-1^)** | ***Left**** | PWS | 0.46 ± 0.15 | 0.40 ± 0.15 | 0.51 ± 0.11 | **Condition:** BOOT < NORM (*p* = 0.028) |  |
|  |  | 3 km/h  4 km/h  5 km/h | 0.28 ± 0.10  0.38 ± 0.12  0.49 ± 0.11 | 0.23 ± 0.09  0.31 ± 0.11  0.43 ± 0.11 | 0.34 ± 0.11  0.44 ± 0.10  0.55 ± 0.11 | **Condition:** BOOT < NORM (*p* = 0.002); EVEN > NORM (*p* = 0.009) & BOOT (*p* = 0.002)  **Speed:** All speeds different to all other speeds (*p* < 0.001) |  |
|  | ***Right*** | PWS | 0.49 ± 0.13 | 0.45 ± 0.12 | 0.42 ± 0.10 | N/A |  |
|  |  | 3 km/h  4 km/h  5 km/h | 0.32 ± 0.16  0.43 ± 0.15  0.52 ± 0.13 | 0.30 ± 0.12  0.38 ± 0.12  0.48 ± 0.10 | 0.27 ± 0.09  0.36 ± 0.10  0.47 ± 0.11 | **Speed:** All speeds different to all other speeds (*p* < 0.001) |  |
| **W_knee_  (% W_tot_)** | ***Left**** | PWS | 33 ± 5 | 31 ± 6 | 36 ± 4 | **Condition:** EVEN > BOOT (*p* = 0.003) |  |
|  |  | 3 km/h  4 km/h  5 km/h | 29 ± 6  31 ± 5  34 ± 3 | 26 ± 5  28 ± 6  32 ± 4 | 34 ± 6  35 ± 4  36 ± 4 | **Condition:** EVEN > NORM (*p* < 0.001) & BOOT (*p* = 0.005); BOOT < NORM (*p* < 0.001)  **Speed:** 5 km/h > 3 km/h (*p* = 0.016) & 4 km/h (*p* = 0.032) |  |
|  | ***Right*** | PWS | 36 ± 7 | 47 ± 7 | 46 ± 6 | **Condition:** BOOT (*p* = 0.003) & EVEN (*p* < 0.001) > NORM |  |
|  |  | 3 km/h  4 km/h  5 km/h | 31 ± 7  34 ± 5  36 ± 4 | 42 ± 8  44 ± 7  48 ± 7 | 42 ± 7  44 ± 6  47 ± 5 | **Condition:** BOOT & EVEN > NORM (*p* < 0.001)  **Speed:** 5 km/h > 3 & 4 km/h (*p* < 0.001); 4 km/h > 3 km/h (*p* = 0.042) |  |
| **Figure 4** | | | | | | | |
| **W_hip_  (J∙kg^-1^)** | | ***Left*** | PWS | 0.48 ± 0.11 | 0.47 ± 0.11 | 0.51 ± 0.07 | N/A |
|  |  |  | 3 km/h  4 km/h  5 km/h | 0.33 ± 0.06  0.41 ± 0.06  0.51 ± 0.04 | 0.30 ± 0.06  0.40 ± 0.07  0.50 ± 0.08 | 0.34 ± 0.06  0.45 ± 0.06  0.55 ± 0.06 | **Speed:** All speeds different to all other speed (*p* < 0.001) |
|  |  | ***Right*** | PWS | 0.49 ± 0.09 | 0.45 ± 0.12 | 0.44 ± 0.09 | N/A |
|  |  |  | 3 km/h  4 km/h  5 km/h | 0.35 ± 0.10  0.42 ± 0.09  0.50 ± 0.08 | 0.35 ± 0.09  0.42 ± 0.11  0.46 ± 0.08 | 0.32 ± 0.06  0.40 ± 0.07  0.46 ± 0.08 | **Speed:** All speeds different to all other speed (*p* < 0.001) |
| **W_hip_  (% W_tot_)** | | ***Left**** | PWS | 35 ± 4 | 37 ± 4 | 36 ± 3 | **Condition:** BOOT > NORM (*p* = 0.005) |
|  |  |  | 3 km/h  4 km/h  5 km/h | 35 ± 3  35 ± 3  35 ± 3 | 35 ± 5  37 ± 5  37 ± 5 | 35 ± 4  36 ± 3  37 ± 3 | N/A |
|  |  | ***Right*** | PWS | 36 ± 5 | 47 ± 7 | 48 ± 7 | **Condition:** BOOT & EVEN > NORM (*p* < 0.001) |
|  |  |  | 3 km/h  4 km/h  5 km/h | 35 ± 4  34 ± 4  34 ± 4 | 51 ± 7  49 ± 7  47 ± 6 | 52 ± 7  50 ± 6  47 ± 6 | **Condition:** BOOT & EVEN > NORM (*p* < 0.001)  **Speed:** 5 km/h < 3 km/h (*p* = 0.001) & 4 km/h (*p* = 0.004)  **Interaction:** NORM: no difference; BOOT: 5 km/h < 3 km/h (*p* = 0.013) & 4 km/h (*p* = 0.006); EVEN: 5 km/h < 3 km/h (*p* = 0.001) & 4 km/h (*p* = 0.002) |
| *PWS = preferred walking speed. * = discrepancy in main effect of condition between PWS and standardised walking speeds.* | | | | | | |  |
