# Supplementary material for: Exploring mechanical work changes in controlled ankle motion (CAM) boot walking: The effects of gait speed and shoe levelling
Source: PLoS One. 2025 Apr 29;20(4):e0321978. doi: 10.1371/journal.pone.0321978 (PMC12040088; doi:10.1371/journal.pone.0321978)
Supplement: Attachment — Submitted filename: ResponsesToReviewerComments.docx [file pone.0321978.s003.docx]

**Gait speed affects hip and knee joint mechanical work done whilst wearing a controlled ankle motion (CAM) boot**

Author Responses to Reviewer Comments

We would like to thank you for your constructive feedback on our manuscript. We value your comments and suggestions and believe there were in many cases valid. We have acknowledged all comments/recommendations and have offered our responses below in red text. Hopefully, our responses and subsequent manuscript are sufficient to ensure our paper is now acceptable. We feel that these modifications have strengthened the manuscript overall.

Major Comments

Clarity of hypotheses and objectives: It would benefit the reader to have clearer articulation of the hypotheses and objectives. While the article implies an interest in understanding how mechanical work distribution changes with footwear, explicitly stating the primary hypotheses would enhance the clarity of the research aim.

Thank you for this comment. We have amended the final paragraph of our introduction to more clearly articulate the primary hypothesis in our study.

Explanation of Conditions (NORM, BOOT, EVEN): The footwear conditions are referenced using abbreviations (NORM, BOOT, EVEN), but the rationale for choosing these conditions is not entirely clear. Please clarify the differences between the BOOT and EVEN conditions and how these comparisons address the study’s primary research question.

Thank you for this comment. We have added some clarity to the second paragraph of our Introduction to state more clearly what the purpose of an even-up walker is. We have also added two sentences to the end of the Introduction to clarify why including EVEN as a footwear condition is important and clinically relevant, as well as what we might expect given the known impact even-up walkers have on preferred walking speed. Finally, some additional justification has been added to the ‘Data collection’ section of the Materials and Methods.

Interpretation of Findings in Contralateral Limb: In the results section, the contralateral limb shows a higher relative knee contribution in the EVEN condition compared to BOOT and NORM, with significant main effects of speed for both limbs. However, the clinical implications of these findings are not fully explored in the discussion. Consider addressing whether increased contralateral knee work could pose risks for injury or discomfort in individuals using CAM boots.

Thank you for this comment. We agree that this consideration is important. We feel we already discussed the elevated knee joint’s contribution to total mechanical work, and it being a possible explanation for the knee joint being a commonly reported site of pain, in the third paragraph of the discussion. However, we have tried to strengthen this point slightly by discussing the fact that this increased contribution could, over time, pose an injury risk, although we should be wary of too much speculation. We hope that our additional discussion provides more clarity here.

Spatiotemporal Parameters Section: The section on spatiotemporal parameters presents several effects of condition and speed on variables such as stance time, swing time, and step length. To improve coherence, consider linking these findings more explicitly to the main objectives of the study. Are these changes expected when wearing a CAM boot, and how do they contribute to compensatory strategies?

Thank you. We have now referred specifically to spatiotemporal parameters in the final paragraph of the introduction, and we have also developed our interpretation in the discussion. We feel that we did already discuss spatiotemporal data in accordance with the aim of our study somewhat, so have added to this by making a clearer link between these and joint kinetics. We have also discussed how the disagreement in spatiotemporal responses between PWS and standardised speeds is a possible risk for interpretation, especially when discussing the possibility of overuse injuries.

Potential Confounding Variables: The study could benefit from acknowledging potential confounding factors that may influence mechanical work distribution, such as participant variability in strength, walking patterns, or experience wearing CAM boots. Addressing these would add depth to the interpretation of the findings.

Thank you. This suggestion has been implemented in the limitations paragraph of the discussion.

Minor Comments

Abbreviations: Certain abbreviations (e.g., PWS for preferred walking speed) are used inconsistently throughout the text. Ensure all abbreviations are defined upon their first usage and maintain consistency.

Thank you for this comment. We noticed a couple of occasions where Achilles tendon had been spelled out despite us offering an abbreviation (AT) in the opening paragraph of the introduction. This has now been amended on all occasions. The other instances are the use of the abbreviation “CAM” for controlled ankle motion boots, which we have defined in both the abstract and in the introduction (we understand that this is appropriate and standard practice) and “PWS” for preferred walking speed, which we have now also abbreviated in the abstract for consistency. We also amended one occasion in the main text of the manuscript.

Statistical Notation: The notation for statistical values should be standardized across the article. For example, clarify whether you consistently use one decimal point for p-values (e.g., p < 0.001 instead of p = 0.001) and check that all reported F-values include the correct degrees of freedom.

Our *p*-values were always reported to 3 decimal places (e.g., *p* = 0.010 or *p* < 0.001). The cases where we present *p* < 0.001 are cases where this value falls beyond 3 decimal places in the direction of zero (although *p* can never = 0). We understand that it is standard practice to present *p*-values this way. Degrees of freedom have also been added to each instance where an ANOVA *F*-statistic is presented.

Figures and Tables Formatting: Ensure that each figure caption (e.g., Figure 3, Figure 4) briefly summarizes the main result rather than reiterating the same information as in the text. This would improve readability for readers who may reference the figures directly.

Thank you. We have tried to summarise some main findings from each figure/table in their respective captions. Hopefully this improves readability.
